# Signal Transduction Mechanisms for Glucagon-Induced Somatolactin Secretion and Gene Expression in Nile Tilapia (*Oreochromis niloticus*) Pituitary Cells

**DOI:** 10.3389/fendo.2020.629077

**Published:** 2021-02-04

**Authors:** Chaoyi Zhang, Anji Lian, Yue Xu, Quan Jiang

**Affiliations:** Key Laboratory of Bio-Resource and Eco-Environment of Ministry of Education, College of Life Sciences, Sichuan University, Chengdu, China

**Keywords:** glucagon, somatolactin, secretion and gene expression, pituitary, tilapia

## Abstract

Glucagon (GCG) plays a stimulatory role in pituitary hormone regulation, although previous studies have not defined the molecular mechanism whereby GCG affects pituitary hormone secretion. To this end, we identified two distinct proglucagons, *Gcga* and *Gcgb*, as well as GCG receptors, *Gcgra* and *Gcgrb*, in Nile tilapia (*Oreochromis niloticus*). Using the cAMP response element (CRE)-luciferase reporter system, tilapia GCGa and GCGb could reciprocally activate the two GCG receptors expressed in human embryonic kidney 293 (HEK293) cells. Quantitative real-time PCR analysis revealed that differential expression of the *Gcga* and *Gcgb* and their cognate receptors *Gcgra* and *Gcgrb* was found in the various tissues of tilapia. In particular, the *Gcgrb* is abundantly expressed in the neurointermediate lobe (NIL) of the pituitary gland. In primary cultures of tilapia NIL cells, GCGb effectively stimulated SL release, with parallel rises in the mRNA levels, and co-incubation with the GCG antagonist prevented GCGb-stimulated SL release. In parallel experiments, GCGb treatment dose-dependently enhanced intracellular cyclic adenosine monophosphate (cAMP) accumulation with increasing inositol 1,4,5-trisphosphate (IP3) concentration and the resulting in transient increases of Ca^2+^ signals in the primary NIL cell culture. Using selective pharmacological approaches, the adenylyl cyclase (AC)/cAMP/protein kinase A (PKA) and phospholipase C (PLC)/IP3/Ca^2+^/calmodulin (CaM)/CaMK-II pathways were shown to be involved in GCGb-induced SL release and mRNA expression. Together, these results provide evidence for the first time that GCGb can act at the pituitary level to stimulate SL release and gene expression *via* GCGRb through the activation of the AC/cAMP/PKA and PLC/IP3/Ca^2+^/CaM/CaMK-II cascades.

## Introduction

Glucagon (GCG), a 29 amino acid peptide hormone, is derived from the precursor proglucagon, which can be processed into multiple bioactive peptides by proteolytic modifications in a tissue-specific fashion (1). In mammals, only a single proglucagon gene encodes GCG (2). Due to genome duplication, fish have duplicate proglucagon genes that generate multiple mRNA transcripts (3). Although GCG shows very weak insulinotropic activity in fishes, it appears to affect metabolism. GCG treatment elevates plasma glucose (4) and lipolytic action by stimulating triacylglycerol lipase in rainbow trout (5). The actions of GCG are mediated *via* the GCG receptor (GCGR), a seven-transmembrane G protein-coupled receptor, which subsequently activates hepatic adenylyl cyclase to increase cAMP production (6). Activation of the cAMP signaling pathway has a diverse repertoire of effects, including initiation of hepatic glycogenolysis and gluconeogenesis (7), stimulation of triglyceride breakdown (8), and enhancement of hepatic fatty acid oxidation (9). Unlike the studies on hepatic GCG function, the signaling mechanisms for the pituitary actions of GCG are still largely unknown.

Somatolactin (SL) is a hormone expressed in the neurointermediate lobe (NIL) of the teleost pituitary, which belongs to the growth hormone (GH)/prolactin (PRL) family (10). In addition to the primary functions of SL in chromatophores regulation (11) and acid-base balance (12–14), the role of SL as an anti-obesity hormone has long been suggested. In gilthead sea bream, exogenous SL treatment inhibits hepatic lipogenic enzymes and stimulates lipid mobilization (15). In contrast, the SL-deficient mutant of medaka significantly increased hepatic triglycerides and cholesterol (11). The involvement of SL in lipid metabolism has also been implied by studying the “cobalt” variant of rainbow trout, which lacks most of the NIL of the pituitary (16). The insulinotropic effect of arginine treatment has a concurrent stimulatory effect on plasma SL and GCG in gilthead sea bream (17). Despite this evidence that links SL and GCG to lipid metabolism, the relevance of the GCG on the lipolytic effect of SL remains unclear. GCG has recently been implicated in the stimulation of pituitary hormone secretion, according to several lines of evidence. First, binding sites for GCG have been identified in the pituitary of goldfish (18), chicken (19), and rat (20). Second, a positive correlation between circulating levels of GCG and GH (21) and administration of GCG induces GH secretion in mammals (22–24). Finally, GCG directly induces the luteinizing hormone (LH) (25) and corticotropin secretion (26) in humans. These findings trigger the possibility that GCG can act on multiple pituitary cell types. However, it remains unclear how SL induced by GCG works, and the potential molecular mechanisms involved are unknown at the pituitary level.

Nile tilapia (*Oreochromis niloticus*) is a global aquaculture species (27). The information regarding the functionality and signaling property of the GCG/GCGR system is limited in tilapia. This limitation prevents our comprehensive understanding of the physiological roles of GCG in tilapia. To bridge this gap, the tilapia was chosen as the animal model in our present study. As a first step, the tilapia duplicated *Gcgs* and *Gcgrs* were established by molecular cloning, and the selectivity of the ligand was characterized by using the functional expression of tilapia GCGR in the human embryonic kidney 293 (HEK293) cells. Moreover, the expression patterns of *Gcgs* and *Gcgrs* in different tissues were examined by quantitative real-time PCR (qPCR). To examine the direct actions of exogenous GCGs on SL expression at the pituitary level, we employed static incubation of NIL cells to study the functional relevance, molecular mechanisms, and signal transduction of SL secretion and gene expression in response to GCGs stimulation. The results presented are the first time to suggest that GCGb could serve as a novel stimulator for SL secretion and gene expression at the pituitary level *via* coupling of AC/cAMP/PKA and PLC/IP3/Ca^2+^/CaM/CaM-KII cascades.

## Materials and Methods

### Animals and Test Substances

Male Nile tilapia (*O. niloticus*) with a bodyweight range of 45-55 g were purchased from local aquaria and maintained in aerated 300-liter aquaria at 28°C under 12 h dark/12 h light photoperiod. During tissue sampling, the fish were sacrificed by spinosectomy after anesthesia with 0.05% MS222 (Sigma, St Louis, MO) according to the procedures approved by the Animal Ethics Committee of Sichuan University. The tilapia and human GCGs and the des-His1-[Nle9-Ala11-Ala16] GCG antagonist were synthesized by GL Biochem (Shanghai, China). The GCG antagonist could block the action of fish glucagon (18). Synthetic peptides were dissolved in double-distilled deionized water and stored frozen in small aliquots at -80°C as 0.1 mM stocks. Pharmacological agents, including the adenylyl cyclase inhibitor MDL12330A, a PKA-specific inhibitor H89, a selective activator of adenylyl cyclase, a cAMP analog 8-Bromo-cAMP, a non-selective phosphodiesterase inhibitor IBMX, a PLC activator m-3M3FBS, a PLC inhibitor U73122, a selective IP3 receptor antagonist, PKC inhibitors GF109203X and calphostin C, a VSCC blocker nifedipine, a SERCA inhibitor thapsigargin, a CaM antagonist calmidazolium, and a CaM-dependent protein kinase-II (CaMK-II) inhibitor KN62 were obtained from Tocris (Ellisville, Missouri, USA). These pharmacological inhibitors with similar doses have been shown to inhibit SL release (28) and gene expression (29) in fish. Pharmacological stocks were prepared as 10 mM in dimethyl sulfoxide (DMSO). The final dilutions of DMSO were less than 0.1% and had no effects on SL release (28) and gene expression (29) in fish pituitary cells.

### Molecular Cloning of Tilapia Gcga, Gcgb, Gcgra, and Gcgrb

Based on the genomic sequences of tilapia *Gcgs* and *Gcgrs*, gene-specific primers were designed, and 5’/3’-RACE was conducted using a GeneRacer Kit (Invitrogen) and sequenced by ABI3100 Genetic Analyzer (BGI, Shanghai, China). The full-length cDNAs for *Gcga* (GenBank no. MT488303) and *Gcgb* (GenBank no. MT488304) were isolated from the tilapia muscle using the RACE method. Besides *Gcgs*, tilapia *Gcgra* (GenBank no. MT488305) and *Gcgrb* (GenBank no. MT488306) were also cloned from the liver and hypothalamus, respectively, using the RACE method. The amino acid sequences of tilapia *Gcgs* and *Gcgrs* were aligned with that of other species by using the DNAMAN programs (Lynnon Biosoft, Quebec, Canada). The motif Scan program was used to predict conserved protein motifs (http://myhits.isb-sib.ch/cgi-bin/motif_scan).

### Functional Expression of Tilapia GCGRa and GCGRb in HEK293 Cells

The open reading frames (ORFs) of tilapia *Gcgra* and *Gcgrb* were subcloned in the pcDNA3.1 (+) expression vector (Invitrogen) using high-fidelity Taq DNA polymerase (TOYOBO, Japan) and then HEK293 cell lines with stable expression of pcDNA3.1-GCGRa, and pcDNA3.1-GCGRb, respectively, were established in the presence of 500 µg/mL Zeocin (Invitrogen). The expression of *Gcgra* and *Gcgrb* transcripts in these stably transfected cell lines was confirmed by qPCR, and the colonies with the same levels of *Gcgra* and *Gcgrb* mRNA expression were selected for subsequent studies. After that, HEK293 cells were co-transfected with the cAMP response element (CRE)-luciferase reporter plasmid, and Renilla luciferase plasmid (pTK-RL) (Promega, Madison, WI), encoding the Renilla luciferase protein, as the internal control using Lipofectamine in Opti-MEM (Invitrogen). After transfection, the cells were incubated overnight at 37°C in Dulbecco’s modified Eagle medium (DMEM) with 10% fetal bovine serum followed by GCGs treatment for another 24 h. After removing the culture medium, HEK293 cells were rinsed with ice-cold phosphate-buffered saline (PBS) and lysed by passive lysis buffer (Promega). The luciferase activity of cellular lysates was determined using a Dual-Glo luciferase assay kit (Promega), and the signal was read using a microplate reader (Fluostar OPTIMA, BMGLabtech). The luciferase activities in each treatment group were expressed as relative fold increase compared with the control group.

### Tissue Distribution of Tilapia Gcga, Gcgb, Gcgra, and Gcgrb

The tissue distributions of *Gcgs* and *Gcgrs* were examined using qPCR. The selected tissues included fat, hypothalamus, intestine, kidney, liver, muscle, pituitary, stomach, and testis. To detect *Gcgra* and *Gcgrb* mRNA expression at the pituitary level, the NIL and pars distalis (PD) of individual pituitaries were isolated by manual dissection under a stereomicroscope as described previously (30). Total RNA was isolated from different tissues of the six male tilapia using TRIzol reagent (Invitrogen) followed by chloroform/isopropanol extraction. RNA was quantified using NanoDrop 1000 spectrophotometer (Thermo Scientific), and 1 µg of total RNA was reverse transcribed using iScript™ Reverse Transcription Supermix (Bio-Rad). qPCR assays were performed on a Mastercycler^®^ ep realplex system (Eppendorf) and analyzed using the software Realplex 2.2 (Eppendorf). qPCR reaction was conducted with a SYBR Select Master Mix kit (Invitrogen) using the primers specific for tilapia *Gcga* [forward primer: 5’ GACGAGCCTGTGGAGTTGTC 3’ and reverse primer: 5’ GCAGCACCACTCCTCTTGTT 3’], *Gcgb* [forward primer: 5’ TCATCATTCAAAGCAGCTGGCA 3’ and reverse primer: 5’ CGTGGCGTCTCCCATTCCTT 3’], *Gcgra* [forward primer: 5’ TGGATCATACGCGCTCCGAT 3’ and reverse primer: 5’ TCGACTTAGCCAACCGGAAC 3’], *Gcgrb* [forward primer: 5’ CCGCTCATATTTGTGTTGCCAT 3’ and reverse primer: 5’ CGGATAATCCACCAATATCCCA 3’]. The qPCR conditions were the following: 2 min incubation at 95°C, 35 amplification cycles (95°C for 30 s, 56°C for 30 s, and 30 s at 72°C, with fluorescence signal detection at the end of each cycle), followed by melting curve of the amplified products obtained by ramped increase of the temperature from 55 to 95°C to confirm the presence of single amplification product per reaction. As an internal control, qPCR for β-actin was conducted using the primers specific for β-actin [forward primer: 5’ GTGATGGTGGGTATGGGT 3’ and reverse primer: 5’ GGCAACTCTCAGCTCGTT 3’]. In these experiments, β-actin was used as an internal control for normalizing genes of interest, and gene expression levels were analyzed using the 2^−ΔΔCT^ method.

### SL Release and Gene Expression in the Primary NIL Cell Culture

To examine the direct effects of GCG on SL release and gene expression in the pituitary, tilapia NIL cells were prepared by the controlled trypsin/DNase II digestion method as described previously (30). After that, NIL cells were seeded in poly-D-lysine precoated 24-well plates at ~1.5 × 10^6^ cells/mL/well and incubated with test substances for the duration as indicated. After drug treatment, SL release in conditioned medium was immediately determined by the enzyme-linked immunosorbent assay (ELISA) with a detectable range from 0.5 to 500 ng/mL for SL. The assay was optimized in our laboratory by utilizing a polyclonal antibody against tilapia SL (31) and a SL-biotin conjugate (GenScript, Nanjing, China). Meanwhile, the cells were rinsed with cold PBS before being lysed by TRIzol reagent (Life Technologies) to measure SL gene expression. After cDNA synthesis, qPCR assays were performed using the primers specific for tilapia SL (forward primer: 5’ CCCACTCCCTTTGCGACTT 3’ and reverse primer: 5’ TAGCGGTCCAGTGTCGTCT 3’). The same qPCR conditions were maintained as described above, and gene expression levels were analyzed using the 2^−ΔΔCT^ method. In these experiments, β-actin was used as an internal control for normalizing genes of interest. It has been reported that β-actin is used to normalize SL gene expression in tilapia (14), goldfish (32), and red drum (33).

### Measurement of cAMP Production and Inositol 1,4,5-Trisphosphate (IP3) Concentration

The pituitary NIL cells were seeded at a density of ~1 × 10^6^ cells/2 ml/dish in 35-mm dishes precoated with poly-D-lysine and cultured overnight at 28°C. Following overnight incubation, the old medium was replaced with 1.8 mL HHBSA (Hank’s balanced salts solution with 25 mM HEPES, 0.1% BSA) medium (Gibco) supplemented with IBMX (0.1 mM) and incubated for 30 min before adding 0.2 mL of 10 × stock solutions of GCGb at 28°C. The duration of the GCGb treatment was fixed at 30 min. After that, cAMP production was quantified using a cAMP ELISA kit (EIAab Science Co., Ltd, Wuhan, China) as previously described (32). The assay for cAMP had a detection sensitivity range of 40–2,500 pmol/L. The intra-assay coefficient of variation was 10%. In parallel studies, the production of IP3 was determined on NIL cells using general inositol 1,4,5,-trisphosphate (IP3) ELISA kit according to the manufacturer’s protocol (CusaBio, Wuhan, China) with a sensitivity range of 5–1,000 pg/mL and intra-assay coefficient of variation was less than 15%.

### Ca^2+^ Signal Measurements in NIL Cells

Tilapia NIL cells were cultured on poly-D-lysine precoated coverslips (~5 × 10^5^ cells/ml/coverslip) for single-cell Ca^2+^ imaging as described previously (28). Briefly, tilapia NIL cells were preloaded with the Ca^2+^-sensitive dye Fura-2-AM (5 µM, Molecular Probes) using a microspectrophotometry fluorescence-ratio setup equipped with a perfusion system at room temperature for 40 min. After that, the ratiometric measurement of intracellular Ca^2+^ level ([Ca^2+^]i) was measured with excitation wavelengths at 340 and 380 nm using a PTI Epifluorescence Ca^2+^ Imaging System (Photon Technology International, Birmingham, NJ) Ca^2+^ data were expressed as a ratio of fluorescence signals with excitation at 340 and 380 nm, respectively (as “F340/380 Ratio”).

### Western Blot of CaM Expression

Western blot was conducted routinely according to the previous description (34). Briefly, proteins were separated in 10% gel by SDS-PAGE and transferred to PVDF (polyvinylidene difluoride, Millipore) using a Hoefer TE70 Semi-Dry Transfer Unit (Pharmacia, San Francisco, CA). After transfer, the membrane was blocked with 5% non-fat dried milk for 1 h and incubated overnight with the primary antibody against human CaM was obtained from Upstate (1:1,000; Milford, MA) at 4°C. The human CaM antibody has been previously used for CaM measurement in fish pituitary cells (29). After washing blots to remove excessive primary antibody binding, the blots were incubated for 1 h with horseradish peroxidase (HRP)-conjugated secondary antibody. Blots were developed by SuperSignal WestPico chemiluminescent substrate (Pierce) using the FluorChem FC2 detection system (Biozym Scientific). The densitometric values of bands were analyzed by ImageJ software (NIH). The results represent prototypical examples of experiments replicated at least three times.

### Statistics Analysis

For qPCR and ELISA assays, NIL cells were prepared from 30–40 individual fish in each experimental run. For cAMP and IP3 measurements, NIL cells were prepared from ~30 individual fish in each experimental run. For Ca^2+^ signal measurements, NIL cells were prepared from 10–12 individual fish. For the Western blot analysis, NIL cells were prepared from ~30 individual fish in each experimental run. Three biological replicates were analyzed, and four technical repeats were performed per sample. All results are expressed as the mean ± SEM (N = 3) and analyzed with ANOVA followed by post-hoc Bonferroni’s test for multiple comparisons. A value of *P* < 0.05 was regarded as statistically significant. All analyses were performed with GraphPad Prism Software, Inc (San Diego, CA).

## Results

### Molecular Cloning of Tilapia Gcga, Gcgb, Gcgra, and Gcgrb

**Figure 1:**
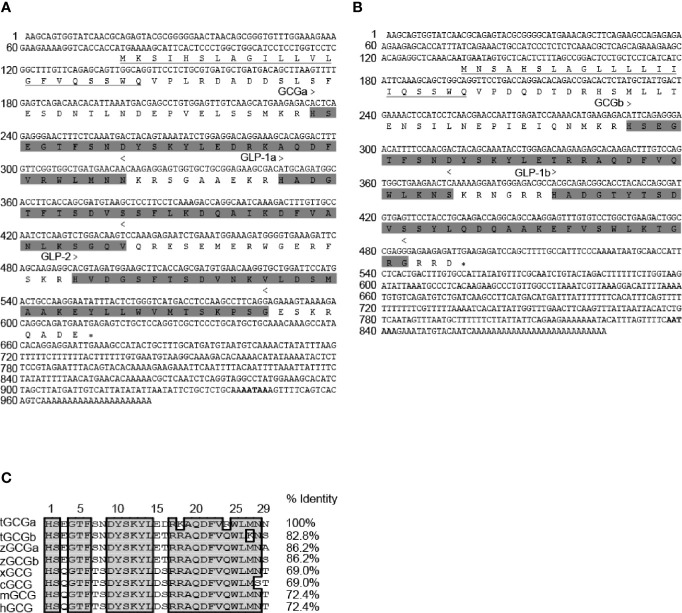
Molecular cloning and sequence alignment of tilapia *Gcga* and *Gcgb*. Nucleotide and deduced amino acid sequences of tilapia **(A)** *Gcga* and **(B)** *Gcgb*. In the amino acid sequence, the putative signal peptides are underlined. GCGs, GLP-1s, and GLP-2 peptides regions are shaded and indicated > to <, and the polyadenylation signal is in bold. An asterisk (*) represents the stop codon. **(C)** Alignment of tilapia GCGs amino acid sequences with the corresponding GCG sequences reported in zebrafish (zGCGa and zGCGb), Xenopus (xGCG), chicken (cGCG), mouse (mGCG), and human (hGCG). Sequence alignment was using the Clustal W algorithm with DNAMAN software. The conserved amino acid residues in these sequences were boxed in gray. The sequences of *Gcgs* for other species were downloaded from the GenBank and/or by searching Ensembl genomes. GenBank accession numbers are as follows: tilapia GCGa (tGCGa, MT488303), tilapia GCGb (tGCGb, MT488304), zebrafish GCGa (zGCGa, NP001008595), zebrafish GCGb (zGCGb, NP001229699), Xenopus GCG (xGCG, NP001079787), chicken GCG (cGCG, NP990591), mouse GCG (mGCG, NP032126), and human GCG (hGCG, NP002045).

The *Gcga* cDNA is 984 bp in size with an ORF of 537 bp encoding a putative 178 amino acids (AAs) preproglucagon. The preproglucagon is composed of a 22 AAs signal peptide followed by a 29 AAs GCGa, a 31 AAs glucagon-like peptide-1 (GLP-1a), and a 33 AAs glucagon-like peptide-2 (GLP-2) (**Figure 1A**). Similarly, the *Gcgb* cDNA is 829 bp in size with an ORF of 363 bp encoding a 120 AAs preproglucagon, containing a putative signal sequence of 21 AAs, mature GCGb sequence of 29 AAs, and GLP-1b of 31 AAs (**Figure 1B**). Analysis of the GCG sequences encoded by *Gcga* and *Gcgb* shows that tilapia GCGs are highly conserved among vertebrate species (69% to 86.2% homology) compared with corresponding peptides. It is noteworthy that tilapia GCGa and GCGb share 82.8% identity at the amino acid level (**Figure 1C**).

**Figure 2:**
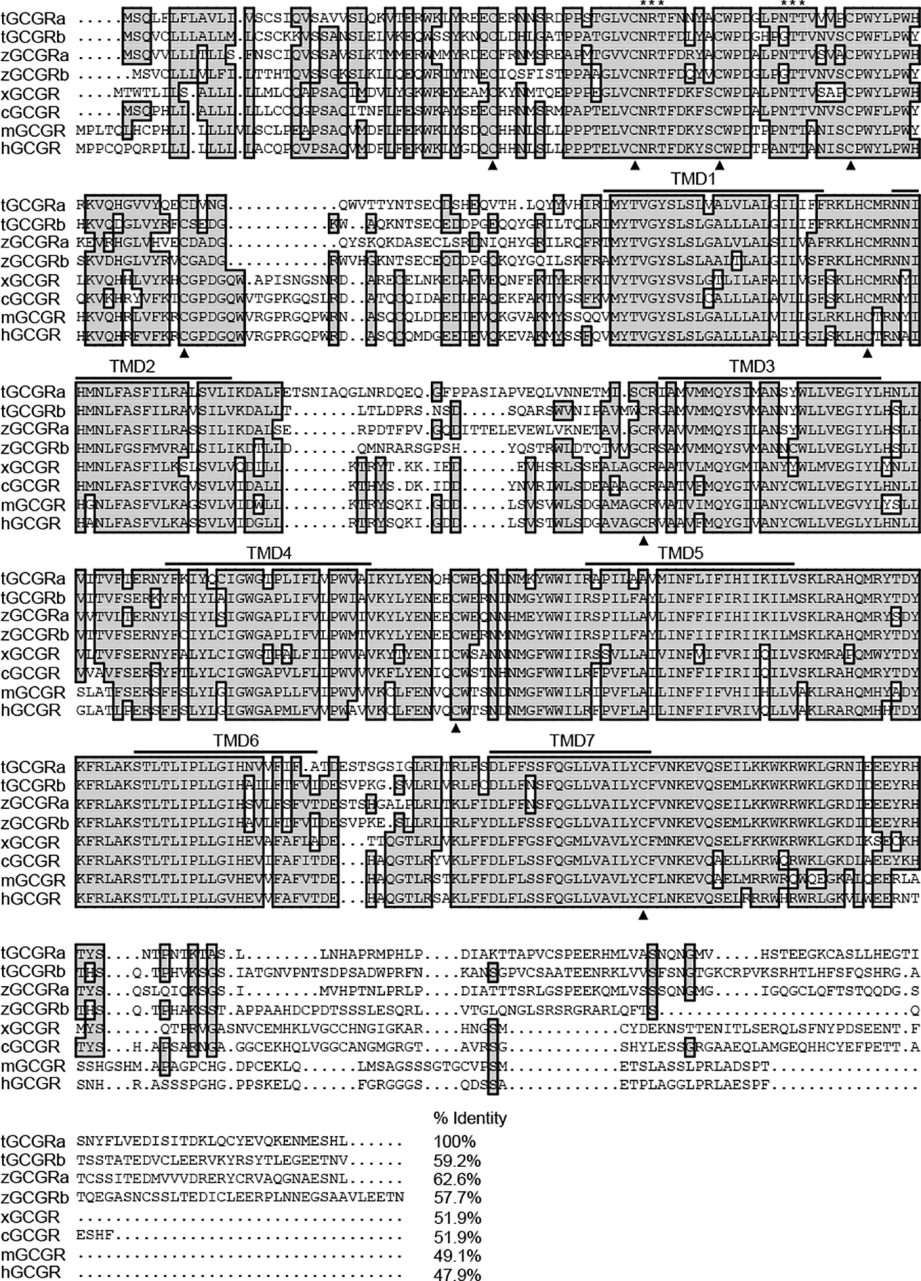
Alignment of tilapia GCGRs amino acid with the corresponding GCGR sequences reported in other species. Conserved amino acids are shaded. Dots (represented by •) are introduced to maximize sequence homology. Putative TMDs are overlined and labeled 1–7. An asterisk (*) represents potential N-linked glycosylation sites, and conserved cysteine residues are indicated as bold triangles. The sequences of GCGRs for other species were downloaded from the GenBank and/or by searching Ensembl genomes. GenBank accession numbers or Ensembl IDs are as follows: tilapia GCGRa (tGCGRa, MT488305), tilapia GCGRb (tGCGRb, MT488306), zebrafish GCGRa (zGCGRa, ENSDARP00000139469), zebrafish GCGRb (zGCGRb, ENSDARP00000013211), Xenopus GCGR (xGCGR, NP001079221), chicken GCGR (cGCGR, NP001094505), mouse GCGR (mGCGR, AAH57988), and human GCGR (hGCGR, EAW89684).

As shown in **Figure 2**, alignment of tilapia GCGRs with other species shows that tilapia GCGRa shares comparatively lower identities with that of tilapia GCGRb (59.2%), zebrafish GCGRa (62.6%), zebrafish GCGRb (57.7%), Xenopus GCGR (51.9%), chicken GCGR (51.9%), mice GCGR (49.1%), and human GCGR (47.9%), respectively. Furthermore, a hydropathy plot of the newly cloned tilapia GCGRs amino acid sequences revealed that typical seven-transmembrane domains (TMDs) are highly conserved among vertebrate species. Besides, the GCGRs sequences contain two conserved N-glycosylation sites (motif NRT and NTT) in the N-terminal domain of all known members of the GCGR, and nine highly conserved cysteine residues are also conserved in all receptors listed.

### Functional Expression of Tilapia GCGRa and GCGRb in HEK293 Cells

**Figure 3:**
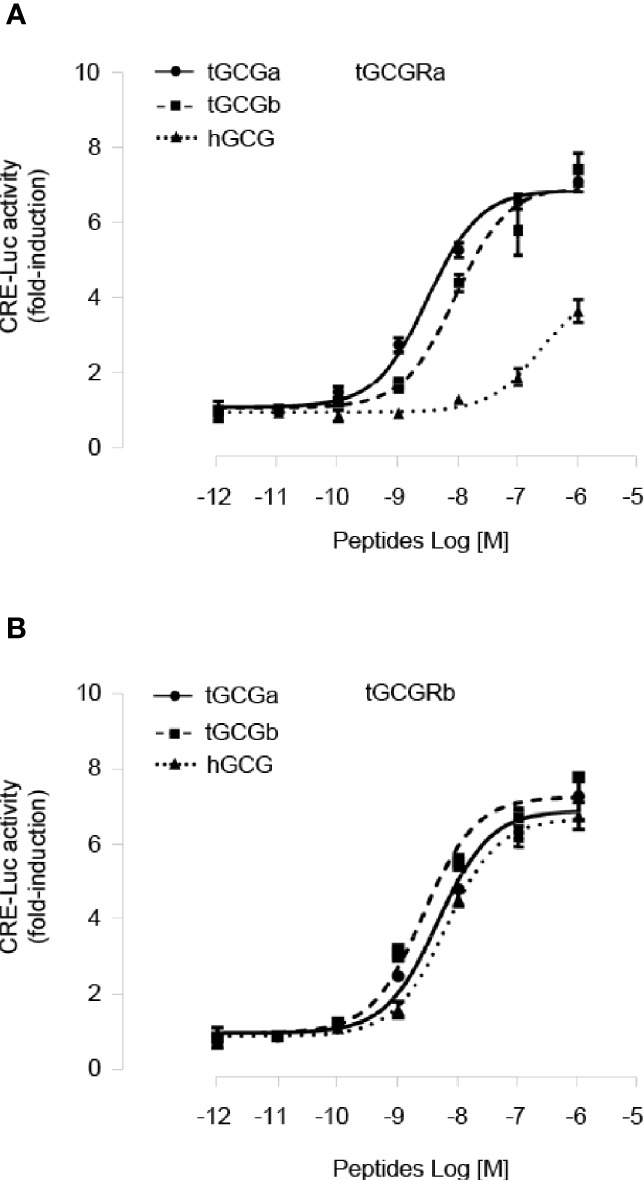
Functional characterization of tilapia GCGRa and GCGRb expressed in HEK293 cells. HEK293 cells with stable expression of tilapia **(A)** GCGRa or **(B)** GCGRb were transfected with CRE-Luc reporter targeting the cAMP pathways and treated for 24 h with increasing doses of the tilapia GCGa, GCGb, and human GCG, respectively. In these experiments, the cotransfection of pTK-RL was used as the internal control, and the data were transformed as a ratio of Renilla luciferase activity in the same sample (as Luc Ratio). All results are expressed as the mean ± SEM (N = 3).

To characterize the ligand selectivity of two tilapia GCG peptides, we determined two GCGs dose-response curves using stable HEK293 cell lines expressing either tilapia GCGRa or GCGRb. As shown in **Figure 3A**, treatment tilapia GCGa and GCGb could dose-dependently elevate cAMP accumulation in HEK293 cells expressing tilapia GCGRa mediated by CRE-luciferase with an EC50 value of 3.04 nM and 8.71 nM, respectively. In contrast, human GCG is much less potent with a much higher EC50 (240 nM) on tilapia GCGRa. In the case of GCGRb activation, both tilapia GCGa (EC50: 4.63 nM) and GCGb (EC50: 2.63 nM) have a similar potency in triggering tilapia GCGRb (**Figure 3B**). Interestingly, we also noted that tilapia GCGRb could be potently activated by human GCG at a slightly higher EC50 of 6.37 nM compared to the tilapia GCGs treatment.

### Tissue Expression of Gcga, Gcgb, Gcgra, and Gcgrb in Tilapia

**Figure 4:**
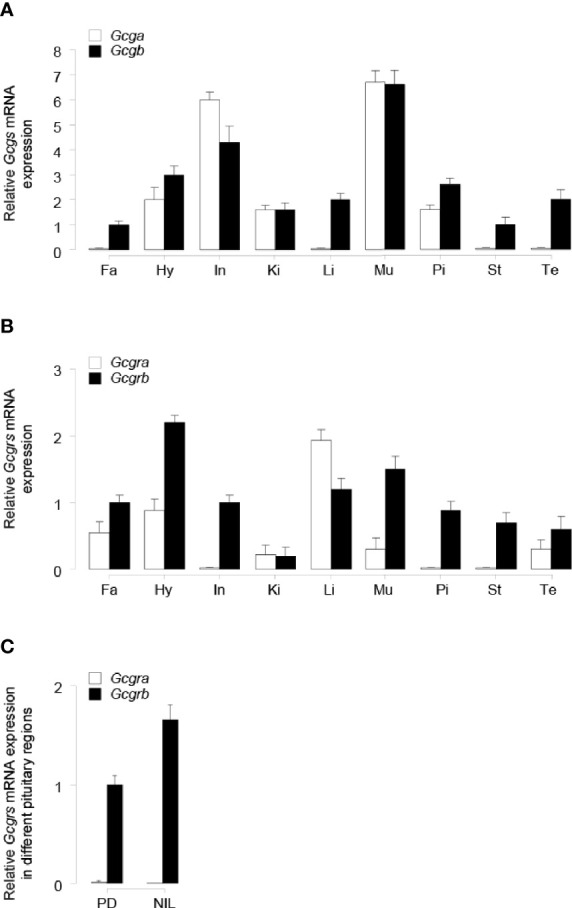
qPCR assay of tilapia **(A)** *Gcgs*, and **(B)** *Gcgrs* mRNA levels in various tissues. The tissues tested in the present study are as follows: Fa, fat; Hy, hypothalamus; In, intestine; Ki, kidney; Li, liver; Mu, muscle; Pi, pituitary; St, stomach; Te, testis. **(C)** Expression profiles of tilapia *Gcgra* and *Gcgrb* in different pituitary regions, including the pars distalis (PD) and the neurointermediate lobe (NIL) region. The error bar for *Gcgra* transcripts in NIL cells was too small to be shown on the plotted scale. The transcripts levels of target genes were normalized by that of β-actin and expressed relative to the fat *Gcgb*, *Gcgrb*, and PD *Gcgrb* levels. The data point of each tissue represents the mean ± SEM of six tilapia.

To establish the tissue expression profiles for tilapia *Gcga, Gcgb, Gcgra*, and *Gcgrb*, qPCR assays were performed in selected tissues. Transcript signals for *Gcga* were detected predominantly in the intestine and muscle, to a lower extent in the hypothalamus, kidney, and pituitary, but with the extremely weak signal detected in the fat, liver, stomach, and testis (**Figure 4A**). Unlike *Gcga*, *Gcgb* transcripts were detected in all the investigated tissues, and the highest expression level of *Gcgb* was found in the muscle (**Figure 4A**). The expression of *Gcgra* mRNA was detected abundantly in the liver, moderately to low in the fat, hypothalamus, kidney, muscle, and testis, and very low mRNA levels could be observed in the intestine, pituitary, and stomach (**Figure 4B**). In contrast, the highest expression levels of tilapia *Gcgrb* existed in the hypothalamus, followed by the muscle, liver, fat, intestine, stomach, pituitary, and testis, and at the lowest level of the kidney (**Figure 4B**). To further provide the anatomical basis for GCG peptides regulation of pituitary SL expression, expression profiles of *Gcgra* and *Gcgrb* in the different parts of the tilapia pituitary were manually dissected under a stereomicroscope and examined by qPCR. As shown in **Figure 4C**, *Gcgra* mRNA was barely expressed in the PD and NIL regions. In contrast, *Gcgrb* transcripts were fairly abundant in the PD region, with the highest expression levels in NIL.

### GCGs Stimulation of SL Release and Gene Expression

**Figure 5:**
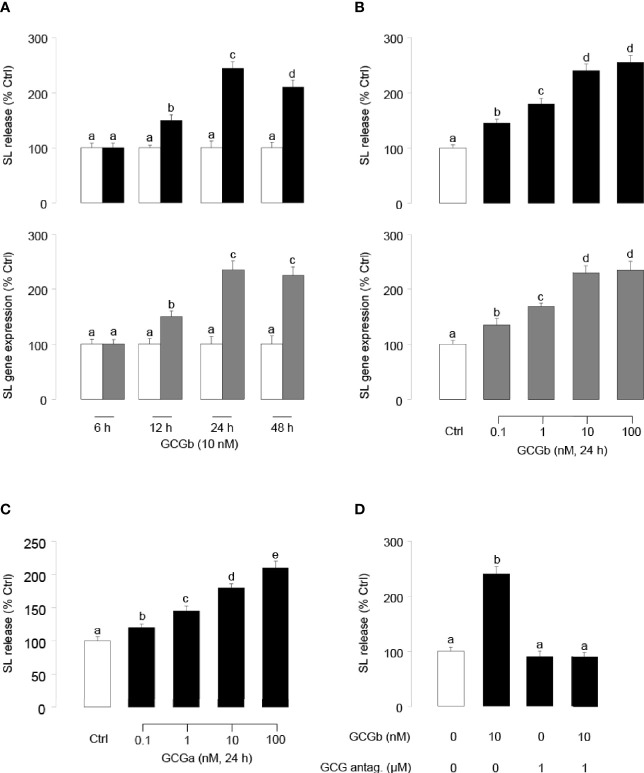
Up-regulation of SL release and gene expression in tilapia NIL cells by GCGs. **(A)** Time course of tilapia GCGb treatment on SL release (upper panel) and mRNA expression (lower panel) in tilapia NIL cells. In these studies, NIL cells were incubated with GCGb (10 nM) for the duration as indicated. **(B)** Dose-dependence of GCGb treatment on SL release (upper panel) and mRNA expression (lower panel) in primary cultured tilapia NIL cells. NIL cells were routinely challenged for 24 h with increasing levels of GCGb (0.1–100 nM). **(C)** Parallel experiment using increasing doses of GCGa as the stimulant. NIL cells were routinely challenged for 24 h with increasing levels of GCGa (0.1–100 nM). **(D)** Effects of GCG antagonist on GCGb-stimulated SL release in NIL cells. The NIL cells were incubated for 24 h with GCGb (10 nM) in the presence or absence of GCG antagonist (1 µM). All results are expressed as the mean ± SEM (N = 3) and different superscript letters in each column show significant differences (*P* < 0.05, ANOVA followed by a Bonferroni’s test).

To examine the direct effects of tilapia GCGb on SL release and gene expression at the pituitary level, time-course studies were conducted to investigate the temporal effects of GCGb on SL release and gene expression using primary cultures of tilapia NIL cells. As shown in **Figure 5A**, GCGb could increase SL release and mRNA expression in time-related manners, and the maximal stimulations on SL release and mRNA expression were observed at 24 h. At a fixed time point, NIL cells challenged with increasing levels of GCGb (0.1–100 nM) for 24 h resulted in concentration-related increases in SL release and mRNA expression (**Figure 5B**). The minimal effective dose for GCGb to stimulate SL release and mRNA expression could be noted at 0.1 nM, respectively, while the maximal responses were noted in the 10- to 100-nM dose range. In parallel studies, GCGa treatment could mimic GCGb-induced stimulation of SL secretion using increasing doses of GCGa (**Figure 5C**). To further confirm the action of GCGb, we incubated primary NIL cell cultures for 24 h with GCGb in the presence or absence of the GCG antagonist (1 µM). In this case, GCGb consistently elevated SL release in tilapia NIL cells, whereas this stimulatory effect could be blocked by simultaneous treatment with GCG antagonist (**Figure 5D**).

### AC/cAMP/PKA Pathway in GCGb-Stimulated SL Release and Gene Expression

**Figure 6:**
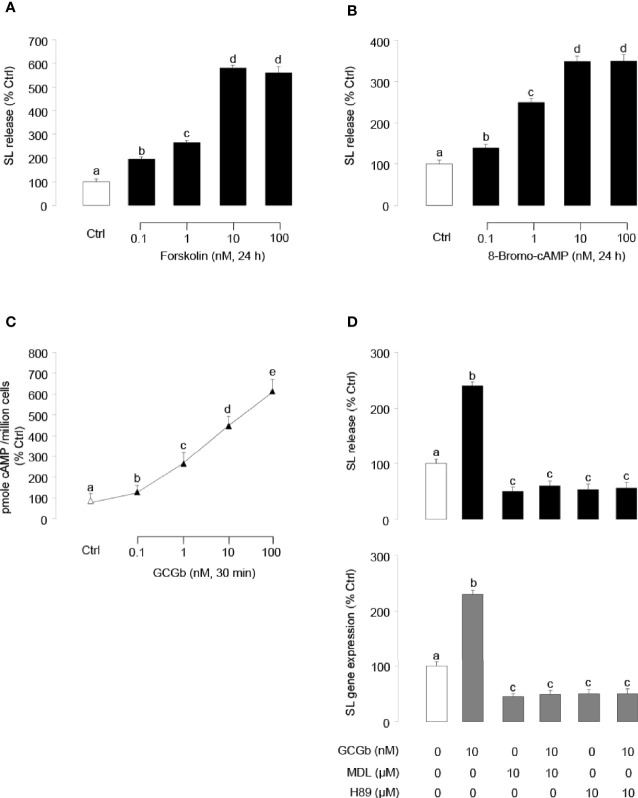
Functional role of AC/cAMP/PKA-dependent mechanisms in SL release and gene expression. **(A, B)** Effects of activating cAMP-dependent cascades on SL release. NIL cells were treated for 24 h with increasing doses of **(A)** the AC activator forskolin (0.1–100 nM) or **(B)** the membrane-permeant cAMP analog 8-Bromo-cAMP (0.1–100 nM). **(C)** Effects of increasing concentrations of GCGb (0.1–100 nM) on total cAMP production in NIL cells. These data were normalized as a function of cell number (as “picomole cAMP produced/million cells”). **(D)** Effects of MDL12330A and H89 on GCGb-induced SL release (upper panel) and mRNA expression (lower panel). NIL cells were incubated for 24 h with GCGb (10 nM) in the presence or absence of the AC inhibitor MDL12330A (MDL, 10 µM), or PKA inhibitor H89 (10 µM). All results are expressed as the mean ± SEM (N = 3) and different superscript letters in each column show significant differences (*P* < 0.05, ANOVA followed by a Bonferroni’s test).

Similar to the results of GCGb treatment, incubating tilapia NIL cell cultures with forskolin (**Figure 6A**) or 8-Bromo-cAMP (**Figure 6B**) dose-dependently stimulated SL release. To examine the possible involvement of cAMP in GCGb actions, the effects of GCGb on cAMP production were tested in NIL cells. As shown in **Figure 6C**, increasing doses of GCGb could result in the increased production of the cAMP of tilapia NIL cells. Since the GCG-induced increase in intracellular cAMP concentration results in the activation of PKA (6), we further employed the AC inhibitor MDL12330A and PKA-specific inhibitor H89 to investigate the role of AC and PKA in mediating the effects of GCGb on SL release and gene expression. Incubating NIL cells with AC inhibitor MDL12330A or PKA inhibitor H89 blocked the basal levels of SL release and gene expression, as well as the GCGb-induced levels (**Figure 6D**).

### PLC/IP3/PKC Pathway in GCGb-Stimulated SL Release and Gene Expression

**Figure 7:**
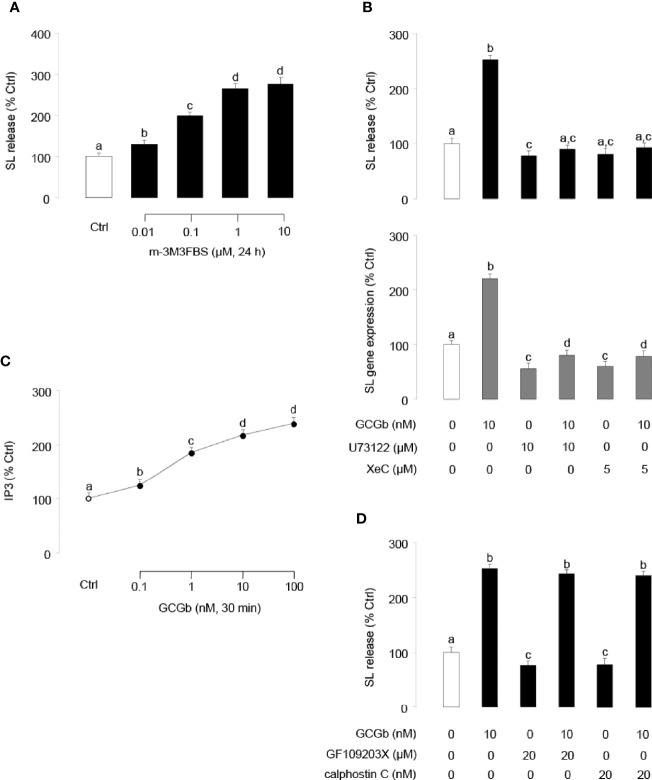
Functional role of PLC/IP3/PKC-dependent mechanisms in SL release and gene expression. **(A)** Effects of increasing doses of the PLC activator m-3M3FBS (0.01–10 µM) on SL release in NIL cells. **(B)** Effects of PLC inhibitor U73122 and IP3 receptor antagonist xestospongin C (XeC) on GCGb induction of SL release (upper panel) and gene expression (lower panel). NIL cells were exposed to GCGb (10 nM) for 24 h with or without simultaneous treatment of the PLC inhibitor U73122 (10 µM) and IP3 receptor antagonist XeC (5 µM). **(C)** Effects of increasing concentrations of GCGb (0.1–100 nM) on IP3 production in NIL cells. **(D)** Effects of PKC inhibition on GCGb-induced SL release. NIL cells are incubated for 24 h with GCGb (10 nM) in the presence or absence of the PKC inhibitors GF109203X (20 µM) and calphostin C (20 nM). All results are expressed as the mean ± SEM (N = 3) and different superscript letters in each column show significant differences (*P* < 0.05, ANOVA followed by a Bonferroni’s test).

To investigate the involvement of PLC in GCGb-induced SL expression, NIL cells were challenged with the PLC activator m-3M3FBS. As shown in **Figure 7A**, PLC activator m-3M3FBS dose-dependently stimulated SL release. In parallel studies, coincubating PLC inhibitor U73122 (10 µM) with NIL cells blocked the GCGb-stimulated SL release and mRNA expression (**Figure 7B**). To confirm the possible involvement of IP3 in GCGb actions, the effects of GCGb on IP3 production were also tested in NIL cells. Increasing the concentration of GCGb was effective in stimulating IP3 production in a dose-dependent manner (**Figure 7C**). To further confirm that IP3 activation is involved in SL expression, pituitary NIL cells were challenged with GCGb (10 nM) in the presence or absence of a selective IP3 receptor antagonist xestospongin C (5 µM). As illustrated in **Figure 7B**, GCGb consistently induced a rise in SL release and gene expression in NIL cells, and the basal and GCGb-induced SL release and mRNA levels were suppressed by xestospongin C. To determine if PKC activation was required for GCGb-stimulated SL release, two different PKC inhibitors GF109203X (20 µM) and calphostin C (20 nM) were tested. Surprisingly, neither compound was able to block GCGb stimulated SL release (**Figure 7D**), indicating that the effect of GCGb on SL release is PKC-independent.

### Ca^2+^-/CaM-Dependent Cascades in GCGb-Stimulated SL Release and Gene Expression

**Figure 8:**
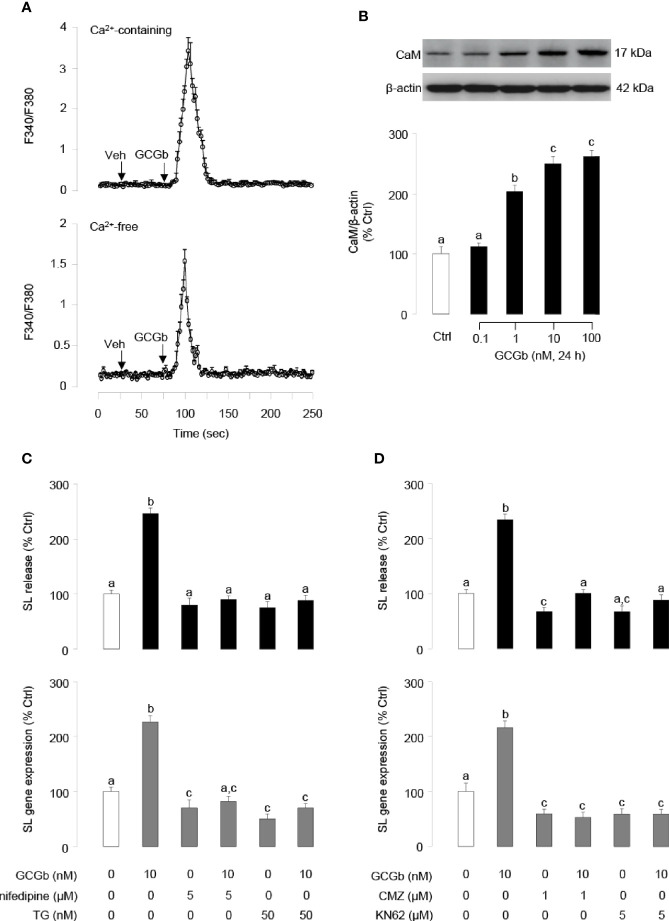
GCGb activation of Ca^2+^/calmodulin (CaM)-dependent cascades in SL release and gene expression. **(A)** The effect of GCGb on total Ca^2+^ signals (upper panel) and [Ca^2+^]i mobilization signals (lower panel) in tilapia NIL cells. In this case, NIL cells were preloaded with the Ca^2+^-sensitive dye Fura-2 and treated with GCGb (10 nM) in a normal culture medium (with 2.5 mM CaCl2) or in a Ca^2+^-free medium supplemented with 0.1 mM EGTA for up to 250 s. **(B)** The stimulatory effects of GCGb (1–100 nM) on CaM protein levels in the NIL cells, measured by western blot and normalized by the internal control, β-actin **(C)** Effects of VSCC blockade and [Ca^2+^]i depletion on GCGb-induced SL release (upper panel) and gene expression (lower panel). The GCGb (10 nM) is applied in the presence or absence of the VSCC blocker nifedipine (5 μM) or the SERCA inhibitor thapsigargin (TG, 50 nM). **(D)** Effects of CaM-dependent cascades blockade on GCGb-induced SL release (upper panel) and mRNA expression (lower panel). NIL cells are challenged with GCGb (10 nM) for 24 h in the presence or absence of the CaM antagonist calmidazolium (CMZ, 1 μM) or the CaMK-II inhibitor KN62 (5 μM). All results are expressed as the mean ± SEM (N = 3) and different superscript letters in each column show significant differences (*P* < 0.05, ANOVA followed by a Bonferroni’s test).

In pituitary NIL cells preloaded with the Ca^2+^-sensitive dye Fura 2, GCGb (10 nM) triggered a rapid rise of both total Ca^2+^ signals (**Figure 8A**, upper panel) and [Ca^2+^]i mobilization (**Figure 8A**, lower panel). In parallel experiments, GCGb could stimulate the cellular content of CaM expression in a dose-dependent manner in NIL cells (**Figure 8B**). To further test the possible involvement of Ca^2+^-dependent pathway in GCGb induction of SL secretion and mRNA expression, NIL cells were challenged with GCGb (10 nM) in the presence of the VSCC blocker nifedipine (5 µM), SERCA inhibitor thapsigargin (50 nM), CaM antagonist calmidazolium (1 µM) and CaM-dependent protein kinase-II (CaMK-II) inhibitor KN62 (5 µM), respectively. Blocking of VSCC and SERCA inactivation could be effective in blocking the stimulatory effect of GCGb on SL release and mRNA expression (**Figure 8C**). Furthermore, GCGb-induced SL release and mRNA expression were abolished by simultaneous treatment with the CaM antagonist calmidazolium or CaMK-II inhibitor KN62 (**Figure 8D**).

## Discussion

The present study was to gain a better understanding of how GCG affects the SL response at the pituitary level. To shed light on the actions of GCG in tilapia pituitary cells, we have cloned a pair of *Gcg* sequences, as well as their functional cognate receptors *Gcgra* and *Gcgrb* in tilapia. Duplicated *Gcg* genes (*Gcga* and *Gcgb*) had previously been characterized in several teleost fish (35) and are thought to be due to the teleost-specific genome duplication (3). As most *Gcg* coding sequences were identified in teleost fish species (36), tilapia *Gcga* generates a transcript that encodes GCGa, GLP-1a, and GLP-2, whereas Gcgb encodes GCGb and GLP-1b, but not GLP-2. In tilapia, His1, Ser2, Asp9, and Ser11 residues are conserved compared to other tetrapods. However, the double substitutions of Ser8 and Ser16 with Asn8, Asp16 (GCGa), and Thr16 (GCGb) were found in the sequences of tilapia GCGs. Although most of the amino acid substitutions in fish GCGs sequences are of similar chemical nature (3), these residues (i.e., Ser8 and Ser16) have been shown to be critical for the formation of the binding pocket of mammalian GCGRs (37, 38).

Because the biological actions of GCG are known to be mediated *via* GCGR, the receptors for tilapia GCGs were also cloned to establish the GCG/GCGR system in the tilapia model. GCGRs have been cloned and characterized from mammals (39, 40), frog (41), avian (19), bony fishes (18, 42). Unlike the only one *Gcgr* found in the higher vertebrates, teleost fish had two *Gcgr* genes due to lineage-specific genome duplications these species experienced (3). Although tilapia GCGRa and GCGRb show a relatively low degree of amino acid sequence identities, two GCGRs identified in tilapia were shown to have the typical features of GPCR containing seven TMDs, two identical N-glycosylation signals, and nine fully conserved cysteine residues. These conserved domains and residues are critical for proper receptor folding, cell surface targeting, and high-affinity ligand binding (38, 43).

Identification of multiple types of *Gcgrs* raises a question of whether a given physiological response is mediated by either a specific receptor or multiple types of receptors. We examined the functional similarity and difference of the two tilapia GCGRs. Functional expression of the two receptors in HEK293 cells demonstrated that tilapia GCGRa could be preferentially activated by tilapia GCGa (~2.9-fold more potent than GCGb). In contrast, the human GCG showed 80-fold less potent activity against tilapia GCGRa than that of tilapia GCGa, which is consistent with the recent findings with the recombinant zebrafish GCGRa (42). Unlike tilapia GCGRa, GCGRb could be activated by two tilapia GCGs and human GCG in the nanomole concentration range. This finding agrees with the described ligand selectivity of the zebrafish, in which GCGRb could also be potently activated by zebrafish GCGs and mouse GCG (42). Functional characterization of GCGR in humans (44) and rats (43) indicated that the residues of Asp64 and Ser80 are involved in ligand binding and receptor activation. In tilapia, both Asp64 and Ser80 residues are conserved in GCGRb, whereas the substitutions of Asp64 and Ser80 with Asn64 and Pro80 present in tilapia GCGRa, which may account for the fact that the human GCG can induce tilapia GCGRb with relatively high potency.

In the present study, *Gcga* and *Gcgb* were found to express predominately in the intestine and muscle, consistent with previous reports that proglucagon is mainly produced in the intestine and muscle of goldfish (45) and lamprey (46). Apart from the intestine and muscle, the differentially abundant transcripts of two GCGs found in tilapia fat, liver, stomach, and testis give insight that *Gcgb* has specific physiological roles. Unlike the *Gcga* expression pattern in tilapia, the highest levels of *Gcgra* transcripts in the liver may indicate that the known primary function of GCG in regulating hepatic glycogenolysis and gluconeogenesis (7). In contrast, relatively high levels of *Gcgrb* mRNA were found in the hypothalamus region. Together with the *Gcgs*/*Gcgrs* signal located in the hypothalamus, it raises the possibility that GCG may be involved in the inhibition of food intake (47) and promotion of body weight loss (48) and may also serve as a functional component of the hypothalamic-pituitary in tilapia. The wide tissue distribution of tilapia *Gcgrs* is similar to that described for the expression of *Gcgr* transcripts in goldfish (18) and mammalian tissues (49, 50).

In our present study, only *Gcgrb* transcripts were relatively abundant in the NIL region, suggesting that GCGb might serve as an autocrine/paracrine factor at the pituitary level. This idea is confirmed by our *in vitro* studies with tilapia NIL cells in which GCGb was found to stimulate SL release and mRNA expression in a time- and concentration-related manner. It is noteworthy that the time of the maximum effect of GCGb on SL release and mRNA are similar, suggesting simultaneous SL release occurred after gene transcription. The pattern of SL response to GCGb was also similar to our previous studies, in which the increase in SL release by pituitary adenylate cyclase-activating polypeptide (10) and insulin-like growth factors (51) also occurred with parallel rises in mRNA expression in carp pituitary cells. Since both GCG subtypes have a similar potency to activate the GCGRb, it is not surprising that GCGa could directly regulate SL secretion, similar to the GCGb. In addition, blockade of the endogenous GCGR by GCG antagonists could suppress the GCGb-induced SL release. These results indicate that GCGb stimulates SL release *via* the GCGRb expressed by NIL cells. These findings are comparable with the reports in which GCG antagonists could inhibit the goldfish GCG-stimulated intracellular cAMP levels in COS-7 cells (18) and block GCG-induced leptin gene expression in goldfish hepatocytes (52). As in mammals, fish GCG was both glycogenolytic and lipolytic (53). On the other hand, it has long been suggested that SL was involved in the regulation of energy homeostasis in fish species. Transgenic overexpression SL markedly stimulated sterol regulatory element-binding transcription factor 1 and acetyl-CoA carboxylase gene expression in zebrafish (54). In line with these observations, SL knockout medaka was associated with increased triglycerides and cholesterol (11). Taken together with this background, the stimulation of pituitary SL expression by GCG may be consistent with significant roles for both peptides in maintaining energy homeostasis in fish. However, our current study did not directly examine the *in vivo* effect of GCG on SL release and gene expression in tilapia, which is a limitation of this investigation. Therefore, future studies are warranted to test the effect of endogenous GCG on SL expression to yield interesting results regarding physiological events.

Although pituitary GCG-mediated molecular signaling pathways remain largely unknown, GCG action in fish hepatocytes is mediated by the AC/cAMP/PKA pathway (52, 55). These findings promote us to investigate similar signaling mechanisms involved in GCGb action in tilapia pituitary cells. In the current studies, the stimulatory effects of GCGb on SL release could be mimicked by AC activator forskolin or by cAMP analog 8-Bromo-cAMP. Furthermore, cAMP production in NIL cells was dose-dependently increased by GCGb treatment. In contrast, both basal and GCGb-induced SL release and gene expression were markedly abolished by blocking AC with MDL 12330A or inactivating PKA by H89. These findings, as a whole, suggest that the AC/cAMP/PKA pathway plays a key role in maintaining both basal levels as well as GCGb induction of SL release and gene expression in tilapia NIL cells. Apart from cAMP-dependent mechanisms, PLC is usually activated by GCGRs coupled to the Gαq family of G proteins (56), and GCG has been shown to induce the production of IP3 in rat hepatocytes (57). Such findings have led us to speculate that these signaling pathways may also be involved in the stimulatory action of GCGb on SL regulation. In tilapia NIL cells, PLC activation by m-3M3FBS was found to increase SL release, and IP3 production was increased by following GCGb stimulation. Consistent with this notion, inhibiting PLC using U73122 and blocking the IP3 receptor with xestospongin C suppressed GCGb-stimulated SL release and gene expression. Of note, PKC inactivation by GF109203X and calphostin C could not suppress GCGb-stimulated SL release, suggesting that PKC is not involved in the downstream signaling after PLC activation. This result was surprising given that previous work in fish hepatocytes had shown that GCGR is also known to couple with the PLC/IP3/PKC pathway (55). The cause for this inconsistency is not readily apparent, but might be due to the physiological differences of specific cell types. Since the Ca^2+^ signal is through activation of the IP3 receptors by IP3 production (58), we suspected that Ca^2+^ mobilization might contribute to GCGb-induced SL secretion and gene expression at the pituitary level. In our present studies, GCGb treatment triggered a transient rise in total Ca^2+^ changes caused by Ca^2+^ entry and [Ca^2+^]i mobilization and increased CaM expression in tilapia NIL cells, suggesting that the Ca^2+^/CaM-dependent signaling pathways might have been activated. The present findings are in agreement with the previous study by Mine et al. (59), where GCG can mediate Ca^2+^ release from both intracellular Ca^2+^ store and extracellular Ca^2+^ influx in rat hepatocytes. This, however, contradicts the study of Xu et al. (60), which documented that extracellular Ca^2+^ was not involved in GCG-induced [Ca^2+^]i mobilization in HEK293 cells. In tilapia NIL cells, induction of SL release and gene expression by GCGb was negated by inhibiting VSCC functionality by nifedipine and depleting [Ca^2+^]i stores using thapsigargin. Besides, inactivating CaM by calmidazolium and antagonizing endogenous CaMK-II using KN62 could prevent SL response to GCGb. These results suggest that [Ca^2+^]e entry through VSCC and [Ca^2+^]i mobilization followed by activation of CaM/CaMK-II cascades are involved in GCGb induction of SL release and mRNA expression at the pituitary level. Our results, taken together, provide novel information on the signal transduction for the GCG/GCGR system of the pituitary gland in basal vertebrates.

**Figure 9:**
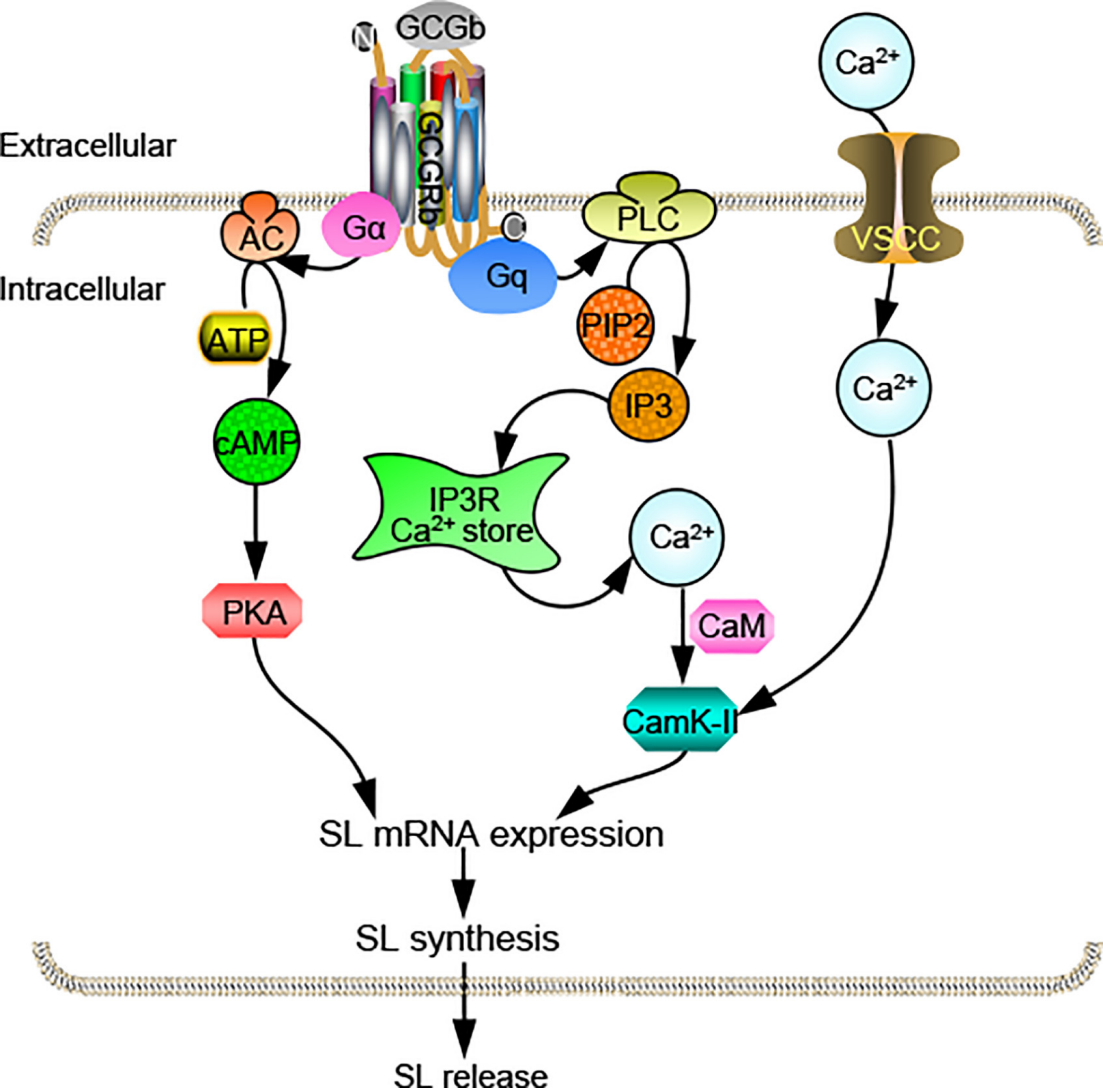
Working model of SL regulation by GCGb induction in tilapia NIL cells. GCGRb activation by GCGb can up-regulate SL release and gene expression *via* stimulation of the AC/cAMP/PKA and PLC/IP3/Ca^2+^/CaM/CaMK-II cascades.

In summary, we obtain two distinct proglucagons, *Gcga* and *Gcgb*, as well as their functional cognate receptors *Gcgra* and *Gcgrb* in tilapia. Functional studies prove that two tilapia GCGRs have similar characteristics in responding to tilapia GCGa and GCGb. qPCR assays reveal that *Gcgs* and *Gcgrs* are overlapping yet distinctly expressed in tilapia selected tissues. Within the anterior pituitary, the only *Gcgrb* is expressed at relatively high abundance in the NIL and likely involved in mediating GCGb-induced SL expression. Using the primary cultures of tilapia NIL cells, we have demonstrated that GCGb can induce SL release and gene expression *via* GCGRb expressed in NIL cells. This stimulatory effect is mediated through the functional coupling of AC/cAMP/PKA and PLC/IP3/Ca^2+^/CaM-dependent pathways (**Figure 9**). The present study, as a whole, provides novel insights on the mechanism responsible for the effect of GCG on the pituitary hormone release and gene expression in basal vertebrate species. Give that action of GCG in pituitary affecting SL expression is only one of the many actions of this peptide, further investigations on the molecular mechanism of GCG-induced other pituitary hormone expression are clearly warranted.

## Data Availability Statement

The raw data supporting the conclusions of this article will be made available by the authors, without undue reservation.

## Ethics Statement

The animal study was reviewed and approved by the Animal Ethics Committee of Sichuan University.

## Author Contributions

QJ was the PI and grant holder. QJ and CZ were responsible for project planning and data analysis. CZ, AL, and YX were involved in functional studies with tilapia NIL cells and HEK293 cells. Manuscript preparation was done by QJ. All authors contributed to the article and approved the submitted version.

## Funding

The project was supported by grants from the National Natural Science Foundation of China (31772824, 31302165).

## Conflict of Interest

The authors declare that the research was conducted in the absence of any commercial or financial relationships that could be construed as a potential conflict of interest.

## Abbreviations

AC, adenylate cyclase; cAMP, cyclic adenosine monophosphate; PKA, protein kinase A; PLC, phospholipase C; IP3, inositol 1,4,5-trisphosphate; PKC, protein kinase C; CaM, calmodulin; CaMK-II, Ca^2+^/calmodulin-dependent protein kinase II.
